# A Novel Simian Adenovirus Associating with Human Adeno-virus Species G Isolated from Long-Tailed Macaque Feces

**DOI:** 10.3390/v15061371

**Published:** 2023-06-14

**Authors:** Nathamon Kosoltanapiwat, Lia van der Hoek, Cormac M. Kinsella, Jarinee Tongshoob, Luxsana Prasittichai, Michelle Klein, Maarten F. Jebbink, Martin Deijs, Onrapak Reamtong, Kobporn Boonnak, Wathusiri Khongsiri, Juthamas Phadungsombat, Daraka Tongthainan, Phitsanu Tulayakul, Marnoch Yindee

**Affiliations:** 1 Department of Microbiology and Immunology, Faculty of Tropical Medicine, Mahidol University, Bangkok 10400, Thailand; jarinee.pan@mahidol.ac.th (J.T.); wathusiri.kho@student.mahidol.edu (W.K.); 2 Amsterdam UMC, Laboratory of Experimental Virology, Department of Medical Microbiology and Infection Prevention, University of Amsterdam, 1105 AZ Amsterdam, The Netherlands; c.m.vanderhoek@amsterdamumc.nl (L.v.d.H.); c.m.kinsella@amsterdamumc.nl (C.M.K.); michelleklein321@gmail.com (M.K.); maartenfj@gmail.com (M.F.J.); m.deijs@amsterdamumc.nl (M.D.); 3 Wildlife Conservation Division, Protected Areas Regional Office 3 (Ban Pong), Department of National Parks, Wildlife and Plant Conservation, Ministry of Natural Resources and Environment, Ratchaburi 70110, Thailand; joi.joize@gmail.com; 4 Department of Molecular Tropical Medicine and Genetics, Faculty of Tropical Medicine, Mahidol University, Bangkok 10400, Thailand; onrapak.rea@mahidol.ac.th; 5 Department of Immunology, Faculty of Medicine Siriraj Hospital, Mahidol University, Bangkok 10700, Thailand; kobporn.boo@mahidol.ac.th; 6 Department of Viral Infections, Research Institute for Microbial Diseases, Osaka University, Osaka 565-0871, Japan; juthamas@biken.osaka-u.ac.jp; 7 Faculty of Veterinary Medicine, Rajamangala University of Technology Tawan-ok, Chonburi 20110, Thailand; daodaraka@gmail.com; 8 Department of Veterinary Public Health, Faculty of Veterinary Medicine, Kasetsart University, Nakhon Pathom 73140, Thailand; fvetpnt@ku.ac.th; 9 Akkhraratchakumari Veterinary College, Walailak University, Nakhonsithammarat 80161, Thailand; marnoch.yi@wu.ac.th

**Keywords:** metagenomics, VIDISCA, virus discovery, novel adenovirus, long-tailed macaques

## Abstract

Metagenomics has demonstrated its capability in outbreak investigations and pathogen surveillance and discovery. With high-throughput and effective bioinformatics, many disease-causing agents, as well as novel viruses of humans and animals, have been identified using metagenomic analysis. In this study, a VIDISCA metagenomics workflow was used to identify potential unknown viruses in 33 fecal samples from asymptomatic long-tailed macaques (*Macaca fascicularis*) in Ratchaburi Province, Thailand. Putatively novel astroviruses, enteroviruses, and adenoviruses were detected and confirmed by PCR analysis of long-tailed macaque fecal samples collected from areas in four provinces, Ratchaburi, Kanchanaburi, Lopburi, and Prachuap Khiri Khan, where humans and monkeys live in proximity (total *n* = 187). Astroviruses, enteroviruses, and adenoviruses were present in 3.2%, 7.5%, and 4.8% of macaque fecal samples, respectively. One adenovirus, named AdV-RBR-6-3, was successfully isolated in human cell culture. Whole-genome analysis suggested that it is a new member of the species *Human adenovirus G*, closely related to Rhesus adenovirus 53, with evidence of genetic recombination and variation in the hexon, fiber, and CR1 genes. Sero-surveillance showed neutralizing antibodies against AdV-RBR-6-3 in 2.9% and 11.2% of monkeys and humans, respectively, suggesting cross-species infection of monkeys and humans. Overall, we reported the use of metagenomics to screen for possible new viruses, as well as the isolation and molecular and serological characterization of the new adenovirus with cross-species transmission potential. The findings emphasize that zoonotic surveillance is important and should be continued, especially in areas where humans and animals interact, to predict and prevent the threat of emerging zoonotic pathogens.

## 1. Introduction

Long-tailed macaques (*Macaca fascicularis*) are non-human primates (NHPs) native to Southeast Asia. In Thailand, the monkeys can be found living in proximity to human territory, such as temples, and outside or near mountains and villagers’ residences, where they can access sufficient food and are fed by humans [1]. NHPs have been described as the original sources of several viruses that spread globally and caused severe impact on humans. Human immunodeficiency virus 1 (HIV-1) first evolved from simian immunodeficiency viruses (SIVs) of chimpanzees, and Ebola virus was transmitted to humans from wild chimpanzees and gorillas, well-known examples of NHP-borne viruses that crossed to humans via contact with wild animal blood or fluids [2]. In human–monkey cohabitation areas, the animal urine and feces contaminate the environment and could be sources of zoonotic pathogens [1]. Enteric viruses, i.e., norovirus, rotavirus, adenovirus, enterovirus, and astrovirus, have been detected in fecal samples from diarrheal and asymptomatic macaques [3,4,5,6]. These virus species are also detected and known to cause disease in humans. A previous study indicated that interspecies reassortment occurred among rotaviruses from monkeys, cattle, dogs, cats, and humans [7]. Genetic analysis of simian norovirus and adenovirus genomes suggests that recombination occurs between monkey and human viruses [4,8]. The chance for new zoonotic viruses to emerge via genetic recombination or reassortment between human and animal viruses could be increased in the areas where humans and monkeys are in close contact. A survey for new pathogens with zoonotic potential is of importance for prediction and prevention of new emerging diseases according to the One Health concept [9].

Metagenomics is the collective analysis of microbial genomes present in specimens. Sequence-based analysis has been increasingly used for pathogen detection in both disease and environmental samples [10]. While a conventional molecular assay, such as PCR, requires the pathogen genome information for primer design, metagenomics utilizes an advantage of high-throughput target-independent next-generation sequencing (NGS) to retrieve overall microbe sequences in samples without a need for specific primer amplification. Therefore, with effective bioinformatics, the approach can be useful for disease surveillance and discovery of unknown pathogens. Viral metagenomics has demonstrated its usefulness for outbreak investigations of emerging influenza, Ebola, as well as the severe acute respiratory syndrome coronavirus 2 (SARS-CoV-2) in humans [11,12,13]. Given that more than 60% of novel viruses are of zoonotic origin [14], virus surveillance or a study on the animal virome can aid in emerging disease preparedness. Due to their close genetic relation, association with humans, and large colony size which facilitates the maintenance of virus transmission, primates, rodents, and bats have been considered as importance sources of zoonotic viruses [15]. Human-infecting viruses, such as coronaviruses, paramyxoviruses, reoviruses, and picornaviruses, have been reported from those animals, using metagenomics and other molecular and serological techniques [16,17].

Success of virus discovery using metagenomics relies on at least two steps, i.e., sample preparation and data analysis. Compared with host background and other environmental microbe genomes, viral genomes often comprise a very low percentage of genetic material in samples, making viral metagenomics a challenge. Methods have been applied to increase virus yields and decrease non-viral background in sample preparation methodologies, including low-speed centrifugation, membrane filtration, nuclease treatment, and sequence-independent amplification [18]. The first three methods are applied prior to nucleic acid extraction. The latter is performed with extracted nucleic acids using techniques such as sequence-independent single-primer amplification (SISPA), multiple displacement amplification (MDA), and cDNA amplified fragment length polymorphism (cDNA-AFLP) virus-targeted random amplification [19]. “Virus Discovery based on cDNA-AFLP”, or the VIDISCA workflow, has been developed. It is an NGS library preparation method combined with a bioinformatics pipeline aiming for virus discovery [20,21]. The method is based on cDNA synthesis using non-ribosomal RNA (rRNA) hexamer primers, restriction digestion of the dsDNA library with *Mse* I, and low-depth sequencing on the Ion Torrent PGM platform. After retrieval of nucleotide sequences, the data are analyzed using a bioinformatics workflow to identify known and potentially novel viruses. Described in detail elsewhere [21], the virus discovery component of the workflow begins with UBLAST alignment of sequence reads to a reference virus protein database. Aligned reads are then queried against a local copy of the GenBank nucleotide database using BLASTn. On the basis of both searches, reads are binned into likely viruses (virus sequences hit in both searches), likely false positives (a non-viral nucleotide alignment), or potentially novel viruses (viral protein alignment but no nucleotide alignment). For virus discovery, these latter sequences are focused on for further verification. VIDISCA was successfully used in identification of a novel human coronavirus, HCoV-NL63, in a child with acute respiratory disease in 2003 [22]. Subsequently, several novel viruses have been identified using VIDISCA in both human and animal specimens, among them an orthobunyavirus in the CSF of a child with severe encephalopathy [23], a rhabdovirus in the plasma of a nodding syndrome patient [24], parvoviruses in bat blood [25], and flaviviruses in the sera of wild lemurs [26].

In this study, we used the VIDISCA metagenomic workflow to identify putatively novel viruses in asymptomatic long-tailed macaque fecal samples collected from Ratchaburi Province, Thailand. PCR primers specific to VIDISCA fragments of a new astrovirus, enterovirus, and an adenovirus were used to survey for the viruses in macaque samples collected from four provinces (Ratchaburi, Kanchanaburi, Lopburi, and Prachuap Khiri Khan). An adenovirus was also isolated, and the whole genome of the virus was sequenced and analyzed for novelty. Neutralizing antibodies to the adenovirus were surveyed in plasmas/sera of macaques and humans living in the same areas to study its zoonotic potential.

## 2. Materials and Methods

### 2.1. Monkey Fecal Sample Collection and Processing

A total of 187 fecal or rectal swab samples from long-tailed macaques (*Macaca fascicularis*) were used in this study. The samples were collected from four provinces, two in central Thailand (Ratchaburi and Lopburi) and two in western Thailand (Kanchanaburi and Prachuap Khiri Khan). These provinces were included in this study because monkeys are commonly found in these areas. The samples were collected from locations where monkeys live in proximity to human habitations. A cross-sectional study was carried out by collecting samples once at each site.

Thirty-three fecal samples collected from Ratchaburi Province in 2019 were processed for metagenomic study by VIDISCA next-generation sequencing (NGS). The feces were diluted 1:4 in nutrient broth (Oxoid, Thermo Fisher Scientific, Waltham, MA, USA) supplemented with penicillin–streptomycin and antimycotics, vortexed, and centrifuged at 5000× *g* for 10 min. One hundred microliters of supernatant was treated with 20 units of Turbo DNase (Thermo Fisher Scientific) and incubated for 30 min at 37 °C. Then, 900 μL of lysis buffer L6 [27] was added, vortexed, and incubated for 10 min at room temperature. The treated samples were stored at −80 °C until required for further nucleic acid extraction.

After obtaining the metagenomic results, feces (*n* = 97) and rectal swabs (*n* = 90) collected from long-tailed macaques in Ratchaburi, Lopburi, Kanchanaburi, and Prachuap Khiri Khan from 2013–2019 were subjected to molecular analysis for the detection of astroviruses, enteroviruses, and adenoviruses. The 97 fecal samples included the 33 samples used in the metagenomics. Feces were mixed with phosphate-buffered saline (PBS) to constitute a 30% (wt/vol) fecal suspension. Rectal swabs submerged in transport media were mixed by vortexing and pressing of the cotton swab to the wall of the collection tube prior to discarding the swab. Fecal and rectal swab suspensions were centrifuged at 2000× *g* for 10 min at 4 °C to pellet fecal debris. Supernatants were collected and stored at −80 °C until use for nucleic acid extraction or virus culture.

### 2.2. Viral Metagenomics by VIDISCA NGS Analysis

Nucleic acids were manually extracted from the DNase-treated fecal samples by the Boom extraction method [27] and subjected for a first-strand cDNA synthesis by reverse transcription with non-ribosomal hexamers [28] using SuperScript II (Thermo Fisher Scientific). Subsequently, a second-strand synthesis was performed with Klenow polymerase (3′–5′ exo^−^, New England Biolabs, Ipswich, MA, USA) and the double-stranded cDNA was purified by phenol/chloroform extraction and ethanol precipitation. Double-stranded cDNA was digested by *Mse* I (TˆTAA; New England Biolabs) and ligated to an adaptor containing a sample identifier sequence. The library was size selection purified using AMPure XP beads (Beckman Coulter, Brea, CA, USA), then amplified by PCR with adaptor-specific primers using Q5 High-Fidelity DNA Polymerase (New England Biolabs). The amplified libraries were purified using AMPure XP beads to retain DNA with a length between 100 and 400 nucleotides. DNA was quantified by a Qubit fluorometer with the Qubit dsDNA HS Assay Kit (Invitrogen, Carlsbad, CA, USA). Sample libraries were pooled at an equimolar concentration. The average nucleotide length of the library (approximately 250 bp) was determined by an Agilent 2100 Bioanalyzer (Agilent Technologies, Palo Alto, CA, USA) using an Agilent High Sensitivity DNA Analysis kit. The pooled library was then clonally amplified on beads using the Ion Chef System and sequenced on the Ion PGM™ Hi-Q™ System with an Ion 318 Chip (Thermo Fisher Scientific).

Sequenced reads in FASTA format were analyzed using the VIDISCA bioinformatics workflow [21]. The raw sequences were first classified as viruses, prokaryotes, or eukaryotes by Centrifuge v1.0.3 using the pre-built NCBI non-redundant nucleotide Centrifuge index. The reads were separately subjected to a virus discovery analysis. rRNA reads were sorted out using SortMeRNA v2.1, and non-rRNA sequences were sorted by length and clustered using CD-HIT v4.7. Clustered non-rRNA reads were searched against a eukaryotic virus protein database using the UBLAST algorithm in USEARCH v10. Those with significant alignments were further queried against the NCBI nucleotide database using BLASTn, and two group of sequences were identified for further verification, (i) confident viral reads (classified as virus against both the aa and nt databases) and (ii) possible unknown virus reads (classified as virus against the aa database only). Besides the confident and possible unknown viral reads, the workflow also classified sequence reads into groups including putative phages (prokaryotic virus), false positives, mitochondrial DNA, Centrifuge (reads identified by the metagenomic tool Centrifuge), and no hit. The data were summarized and visualized using KronaTools v2.7 and ggplot2. Sequences of the VIDISCA fragments classified in each category were extracted as FASTA files and used for further inspection by BLASTx and for primer design.

### 2.3. Nested PCR and RT-PCR

Viral nucleic acids were extracted from 200 μL of processed fecal/rectal swab samples using a GenUP™ Virus DNA/RNA kit (Biotechrabbit GmbH, Berlin, Germany) according to the manufacturer’s instructions. The nucleic acid was eluted in 60 μL of elution buffer and stored at −80 °C until further use.

For detection of astrovirus and enterovirus, cDNA synthesis was performed using Maxime RT Pre-mix with random primers (iNtRON Biotechnology, Gyeonggi-do, Republic of Korea) with 5 μL of the extracted viral nucleic acids in a total volume of 20 μL. For adenovirus detection, the extracted viral nucleic acids were used as a PCR template. Specific primers for nested PCR were designed based on sequences of VIDISCA fragments and are shown in Table 1. The first-round PCR reaction contained 1× MyTaq HS Red Mix (Bioline, London, UK), 0.4 µM of each forward and reverse primer, and 3 µL of cDNA (for astrovirus and enterovirus) or 1 μL extracted nucleic acids (for adenovirus), in a total volume of 25 µL. The thermal cycling conditions were set as 95 °C for 3 min, followed by 35 cycles of 95 °C for 15 s, 50 °C or 55 °C for 15 s, and 72 °C for 20 s and a final elongation at 72 °C for 10 min. The nested-PCR reaction was prepared using the same reaction condition as the first PCR with 1 μL of the first-round PCR product (diluted 1:10). The thermal cycling conditions were set as 95 °C for 3 min, followed by 35 cycles of 95 °C for 15 s, 50 °C or 55 °C for 15 s, and 72 °C for 15 s and a final elongation at 72 °C for 10 min. PCR products were resolved on a 1.5% agarose gel, stained with GelRed, and visualized under a gel documentation system (Molecular Imager Gel Doc XR imaging system, Bio-Rad, Hercules, CA, USA). The specific annealing temperatures for each primer pair and expected PCR product sizes are shown in Table 1. Nested-PCR products showing DNA bands of the expecting size were sent for BTSeq sequencing at Celemics, Inc. (Seoul, Republic of Korea). The sequencing results were analyzed by a BLAST search and aligned with the sequence of the VIDISCA fragment for which the primers were designed.

### 2.4. Virus Isolation

Virus isolation was performed using Vero and rhabdomyosarcoma (RD) cells [6]. The cells were grown in Dulbecco’s modified Eagle’s medium (DMEM) supplemented with 10% fetal bovine serum (FBS), 1× antibiotic–antimycotic solution, and 1× GlutaMAX (Gibco, Thermo Fisher Scientific) to a confluency of 80–90%. The supernatants of 30% (wt/vol) fecal solutions were filtered through a 0.45 µm pore membrane using a syringe filter. The solutions were diluted five-fold with viral growth medium (DMEM supplemented with 2% FBS, 1× antibiotic–antimycotic solution, and 1× GlutaMAX) and subsequently inoculated onto the cell monolayer in a 96-well tissue culture plate (50 µL/well, 3 wells/sample). The plate was centrifuged at 25 °C for 20 min prior to incubation at 37 °C under a 5% CO_2_ atmosphere for 20 min for virus adsorption. The centrifugation step was repeated before viral growth medium (150 µL/well) was added, and the cells were incubated at 37 °C under a 5% CO_2_ atmosphere. The cells were observed microscopically for any cytopathic effect (CPE) daily for 5 days. CPE was observed as detached round cells compared with cells anchored to the control wells. Virus isolation was performed for three blinded passages. Virus-positive cultures, as determined by the observation of CPE in Vero or RD cells, were collected and stored at −80 °C for further virus propagation for mass spectrometry and whole-genome sequencing studies.

### 2.5. Viral Protein Identification by Mass Spectrometry

Viruses were propagated in RD cell culture. Fifty milliliters of the virus supernatant was collected, and cell debris was removed by centrifugation at 1000× *g* for 15 min at 4 °C. Viruses in the supernatant were concentrated by ultracentrifugation in polycarbonate centrifuge bottles (no. 355603, Beckman Coulter) using a Beckman L7–65 ultracentrifuge (rotor 70.1 Ti) set at 35,000 rpm for 1.5 h at 4 °C. After ultracentrifugation, pellets were mixed with lysis buffer containing 1% NaCl, 1% SDS, and 1% Triton-X to produce a virus protein lysate. The lysate was analyzed by mass spectrometry using liquid chromatography (LC)–MS/MS by a MicroToF Q II mass spectrometer (Bruker, Leipzig, Germany) coupled to an Ultimate 3000 nano-LC system (Dionex, Sunnyvale, CA, USA) [29].

Briefly, the virus protein lysate was size-separated via 12% SDS-PAGE. The gel was stained with the Coomassie brilliant blue G250 solution (Bio-Rad) and cut along its length into 15 pieces. Each gel piece was cut into equal small cubes and separately destained with a destaining solution (50 mM NH_4_HCO_3_, 50% (*v*/*v*) acetonitrile (ACN)). Gel cubes containing proteins were treated with 5 mM dithiothreitol (DTT) and alkylated in 250 mM iodoacetamide (IAM). The gels were then incubated in the dark for 30 min prior to dehydrating with ACN and digesting with trypsin (100 ng/mL). After tryptic digestion, peptides were extracted from the gels using 50% (*v*/*v*) ACN, dried using a vacuum evaporator, and resuspended in 0.1% formic acid. The resuspended peptides were then subjected to the mass spectrometer.

LC-MS/MS raw data files were generated and converted into mascot generic format (.mgf) files using DataAnalysis™ software version 3.4. The .mgf files were searched using the Mascot program version 2.4.1 (Matrix Science, Boston, MA, USA) against the NCBI database. The organism for searching was set as “virus”. The maximum number of missed cleavages was set to 1. Peptide tolerance was set to 1.2 Da, and tandem MS tolerance was set to 0.6 Da. The fixed modification was set to cysteine carbamidomethylation, and the variable modification included methionine oxidation. All reported peptides showed a confidence level more than 95%.

### 2.6. Whole-Genome Analysis

Viral nucleic acids were extracted from virus culture supernatant of AdV-RBR-6-3 using the GenUP™ Virus DNA/RNA kit without addition of RNA carrier. The nucleic acids were transferred to GenTegra^®^-DNA tubes for ambient temperature storage and transport for whole-genome sequencing via an NGS service (Vishuo Biomedical (Thailand) Ltd., Bangkok, Thailand). The NGS library was prepared using a NEBNext^®^ Ultra™ DNA Library Prep Kit for Illumina^®^ (Illumina, San Diego, CA, USA) following the manufacturer’s protocol. Sequencing was performed on the Illumina HiSeq platform in a 2 × 150 bp paired-end (PE) configuration with 1.0 Gb raw data per sample. Base calling was performed by Illumina RTA software on the HiSeq instrument. Raw sequences were processed via the FASTP program for quality control and adapter trimming. The sequence quality assessment was reported by FASTQC and MultiQC tools. Genome assembly was performed using the cleaned sequences by 2 methods: (i) de novo assembly of host-depleted sequences and (ii) referenced genome assembly using candidate genomes retrieved from (i). Removal of host background was performed using Genome Reference Consortium Human Build 38 patch release 13 (GRCh38.p13) as a reference genome. De novo assembly was performed using SPAdes with minimum contig size of 500 bps [30].

Regions absent of sequence coverage by the whole-genome analysis were amplified by PCR and sequenced. Primers were designed based on sequences of the assembled AdV-RBR-6-3 genome, flanking the missing regions (Table S2). PCR was performed as described above with adjusted annealing temperature and extension time. PCR products were sent for BTSeq™ sequencing at Celemics, Inc. (Seoul, Republic of Korea). The sequences were processed in BioEdit 7.2.5 [31] to construct a complete genome of AdV-RBR-6-3. Open reading frames (ORFs) were predicted using the ORFfinder program and verified by BLAST on the NCBI website. The complete genome sequence of AdV-RBR-6-3 was submitted to the GenBank database and received the accession number OQ579036.

### 2.7. Phylogenetic and Recombination Analysis

Nucleotide sequences of the complete genome and hexon, fiber-1, and CR1 genes of AdV-RBR-6-3 were aligned with published reference sequences using ClustalW in BioEdit. Phylogenetic trees were constructed using Molecular Evolutionary Genetics Analysis (MEGA, version 7.0.26) software [32]. The maximum likelihood method with particular models was applied for each gene based on the phylogenetic model analysis. The general time reversible model was used for the complete genome, hexon and CR1. The Hasegawa–Kishino–Yano model was used for fiber-1. Bootstrap resampling analysis of 1000 replicates was used.

Recombination analyses were performed with the complete AdV-RBR-6-3 genome and simian adenovirus genomes retrieved from GenBank using two software, SimPlot v3.5.1 [33] and 3SEQ v1.7 [34]. The sequences were aligned using ClustalW in BioEdit. Similarity plot and bootscan analyses were performed by SimPlot using default parameter settings. The Kimura 2-parameter distance model, in a sliding window of 200 nucleotides, and step size of 20 nucleotides was applied for the similarity plot, and the Kimura 2-parameter distance model and neighbor-joining tree model, in a sliding window of 200 nucleotides, and step size of 20 nucleotides were used for bootscan analysis. In 3SEQ, sequences were aligned, and all sequence triplets were tested using a non-parametric statistic for mosaicism. A mosaic recombination signal indicating that one of the three sequences is a recombinant of the other two parent sequences was obtained with a significant level for a two-breakpoint recombination signal setting at *p* = 0.05.

### 2.8. Virus Neutralization Assay

The CPE-based microneutralization (NT) assay was performed on RD cells in 96-well microculture plates. Test specimens (103 long-tailed macaque plasma and 125 human serum samples) were heat-inactivated at 56 °C for 30 min and serially two-fold diluted with DMEM, starting from a dilution of 1:10. The diluted serum or plasma samples were mixed with an equal volume of a test virus suspension (prepared at a concentration of 1000 TCID_50_/mL) and incubated at room temperature for 1 h. Fifty microliters of virus–serum mixture was transferred in duplicate to wells containing the RD cell monolayer in 150 µL of maintenance medium (DMEM supplemented with 2% fetal bovine serum) and incubated at 37 °C under a 5% CO_2_ atmosphere. CPE was assessed at day 3 post-inoculation under a light microscope. One hundred percent CPE was read in the virus-infected control wells in which all cells were round and detached as a result of virus infection, and no CPE (0%) was read in the uninfected control wells. The neutralizing antibody titer was defined as the highest reciprocal of the serum or plasma dilution that inhibited ≥50% CPE in wells containing the serum–virus mixture compared with wells with uninfected and virus-infected control cells. A titer of 10 or greater was considered positive for NT antibodies [35]. Two independent experiments were performed.

## 3. Results

### 3.1. Detection of Potential Unknown Viruses by Metagenomics

**Figure 1:**
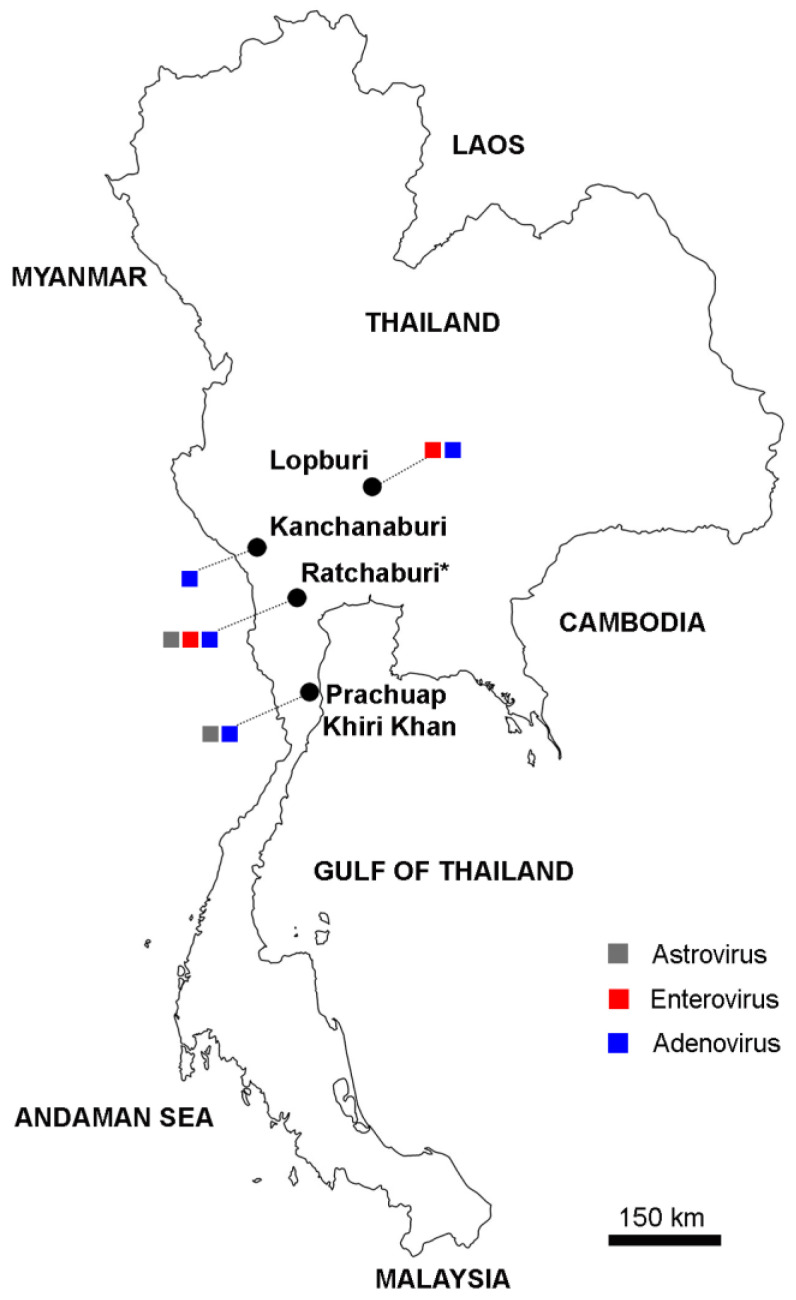
Map of Thailand showing sites of long-tailed macaque fecal sample collection. Provinces where the samples were collected are indicated by black circles. Star (*) indicates Ratchaburi Province. Samples from this region were analyzed by VIDISCA NGS and by virus culture. Colored squares indicate types of viruses detected by PCR in each site. The color labeling for each virus is indicated in the figure.

Of the fecal samples from long-tailed macaques collected in Ratchaburi (Figure 1), 33 were subjected to viral metagenomic analysis using the VIDISCA NGS workflow, generating 37,059 to 82,946 sequence reads per fecal sample. Through the bioinformatic workflow, 10 to 378 sequence reads were identified in each sample as “virus (aa)”, which referred to sequences that had a match in the eukaryotic virus protein database but were unclassified by BLASTn against the NCBI nucleotide database [21]. Those sequence fragments were classified as potentially belonging to unknown viruses according to the VIDISCA platform. By manual inspection and re-confirmation using BLASTn, VIDISCA fragments belonging to a possible unknown astrovirus, enterovirus, and adenovirus were identified in two, three, and one macaque sample, respectively. Subsequently, sequences of the VIDISCA fragments were used for primer design (Table 1). Nested PCR or RT-PCR was performed with the 33 metagenomic samples and the additional macaque fecal samples collected from 4 different areas in Thailand (187 samples in total, Figure 1 and Table 2) to confirm the presence of the VIDISCA-identified putatively new virus sequences in the macaque samples. Astrovirus sequences were detected in macaque feces from Ratchaburi (10.3%) and Prachuap Khiri Khan (2.2%). Nucleotide sequence alignment showed 100% sequence identity between the PCR products and the sequence of the astrovirus-VIDISCA fragment. A BLAST search showed that the sequences were closely related to that of a human-like astrovirus isolated from a macaque, with 83.5% sequence identity (Table S1). Enterovirus sequences were detected in macaque feces from Ratchaburi (12.8%) and Lopburi (19.6%). Nucleotide sequence analysis of the PCR products separated the sequences into two groups based on the site of sample collection. Sequences retrieved from macaques in Ratchaburi showed 97.5–98.7% sequence identity, whereas sequences from Lopburi showed 81.0–83.5% sequence identity compared with the sequence of the enterovirus-VIDISCA fragment. A BLAST search suggested the presence of *Enterovirus A* in these samples, with nucleotide sequence identity of 82.2–88.2% compared with the reference nucleotides in the database (Table S1). Adenovirus sequences were detected in macaque samples from all sites (1.1% to 16.7%) (Table 2). Nucleotide sequence analysis showed 99.3% sequence identity between the macaque sequences and the sequence of the adenovirus-VIDISCA fragment. A BLAST search showed that the sequences were 100% identical to the sequence of AdV-RBR-6-3 (accession no. OQ579036.1) (Table S1). Enterovirus and astrovirus coinfections were detected in two samples from Ratchaburi.

### 3.2. Isolation and Whole-Genome Analysis of Potential New Adenovirus from Macaque Feces

Fecal samples from the Ratchaburi site (*n* = 33) were subjected to virus isolation in Vero and RD cells. Among viruses isolated in RD cells, one virus was isolated from the fecal sample in which VIDISCA metagenomics and nested PCR detected a presence of potential unknown adenovirus (sample no. 6-3). Mass spectrometric analysis by LC-MS/MS confirmed a presence of adenovirus protein in the virus culture supernatant. The top three proteins identified by MS were hexon of human adenovirus (HAdV) F serotype 40, hexon of HAdV-B serotype 7, and hexon of HAdV-B serotype 3, with *p* value < 0.05. The virus was named AdV-RBR-6-3 and was positive in PCR using the VIDISCA-based adenovirus primers (Table 1).

**Figure 2:**
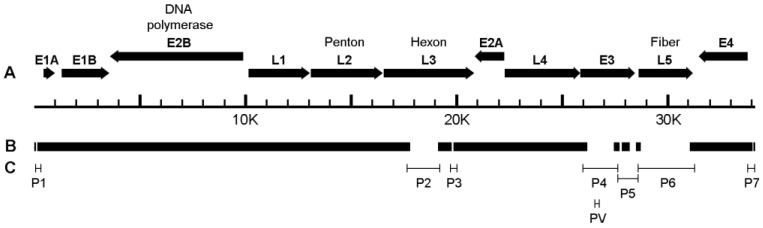
Map of AdV-RBR-6-3 genome assembly. (**A**) The whole genome of AdV-RBR-6-3 is 34,144 bp coding for at least 11 groups of early (E) and late (L) proteins. (**B**) By de novo and reference sequence assembly of NGS contigs, whole genome of AdV-RBR-6-3 was initially retrieved with gaps. (**C**) Primers were designed for PCR and nucleotide sequencing to close the gap regions. Bars in (**C**) represent primer binding and PCR products. P1–P7 are primer names of which sequences are provided in Table S2. PV indicates site of VIDISCA PCR product in E3 region.

Whole-genome sequencing of AdV-RBR-6-3 was performed by NGS. The sequencing result was analyzed by two methods, i.e., de novo assembly and referenced genome assembly. De novo assembly was carried out after the host sequences were removed. As the virus was cultured in a human rhabdomyosarcoma (RD) cell line, human sequences were removed. Contigs resulting from de novo assembly were analyzed by BLAST against the NCBI nucleotide database, and the first three genomes with best matches (minimum alignment >1000 bps, identity percent >85%, and score >1000) were identified and used as reference genomes for reference genome assembly, which were Rhesus adenovirus 53 (KM591903.1), Rhesus adenovirus 55 (MF198449.1), and Simian adenovirus 11 strain P-10 (KP329562.1). The AdV-RBR-6-3 sequences were mapped onto the three-candidate adenovirus reference genomes using bowtie2. Percent mapping values of total sequences on reference genomes were 49.8% for Rhesus adenovirus 53, 32.3% for Rhesus adenovirus 55, and 31.0% for Simian adenovirus 11. Based on the highest %mapping, the Rhesus adenovirus 53 genome sequence was used as a reference for consensus genome assembly. After these two analyses, the AdV-RBR-6-3 genome was obtained with zero-coverage regions mainly in L3 (hexon), E3 (CR1), and L5 (fiber) genes (Figure 2A,B). Conventional PCR and nucleotide sequencing were used to fill in the gaps (Figure 2C and Table S2). After gap fixing, the whole genome of AdV-RBR-6-3 was found to be 34,144 bp. ORFs were identified and the sequence was submitted to GenBank and received accession no. OQ579036. The AdV-RBR-6-3 genome contains at least 33 ORFs in 11 transcription classes (E1A, E1B, E2A, E2B, E3, E4, L1, L2, L3, L4, and L5), encoding structural and non-structural proteins found in mastadenoviruses.

**Figure 3:**
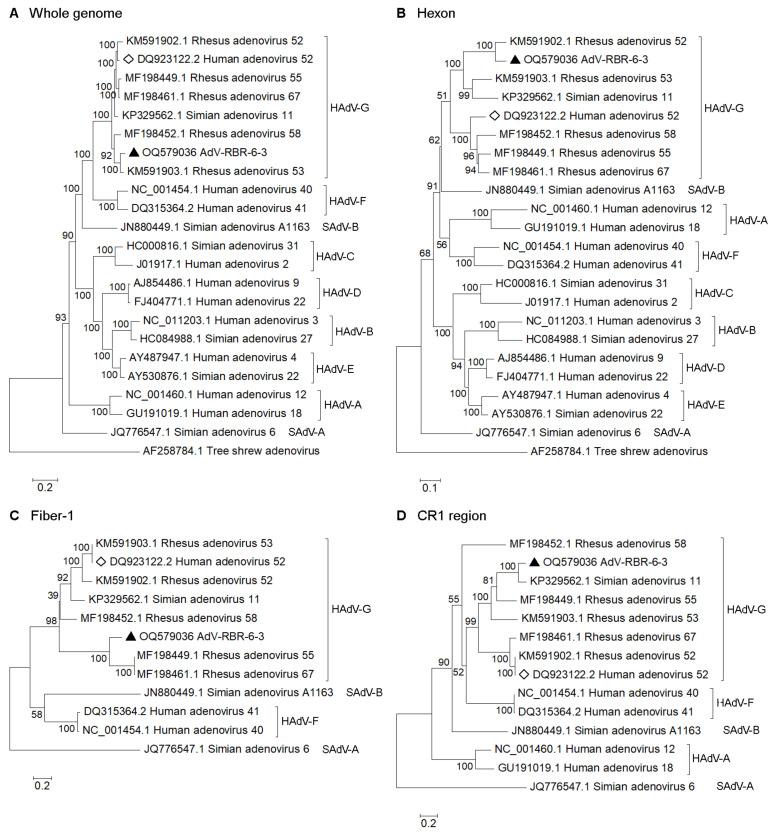
Phylogenetic analysis of (**A**) whole genome, (**B**) hexon, (**C**) fiber-1, and (**D**) CR1 genes of AdV-RBR-6-3 compared with reference sequences of *Human adenovirus A* to *G* (HAdV-A to -G), *Simian adenovirus A* (SAdV-A) and *B* (SAdV-B). Black triangle indicates AdV-RBR-6-3. Diamond indicates Human adenovirus 52, the only member of species HAdV-G isolated from human. Tree shrew adenovirus sequences were used as an outgroup for the whole genome and hexon trees. SAdV-A sequences were used as an outgroup for the fiber-1 and CR1 trees. According to variabilities in fiber and CR1 regions amongst mastadenoviruses, sequences with ≥30% identities were included in the trees. The trees were constructed using the maximum likelihood method with a bootstrap of 1000. Bootstrap values are shown at the node. The bar represents nucleotide substitutions per site.

**Figure 4:**
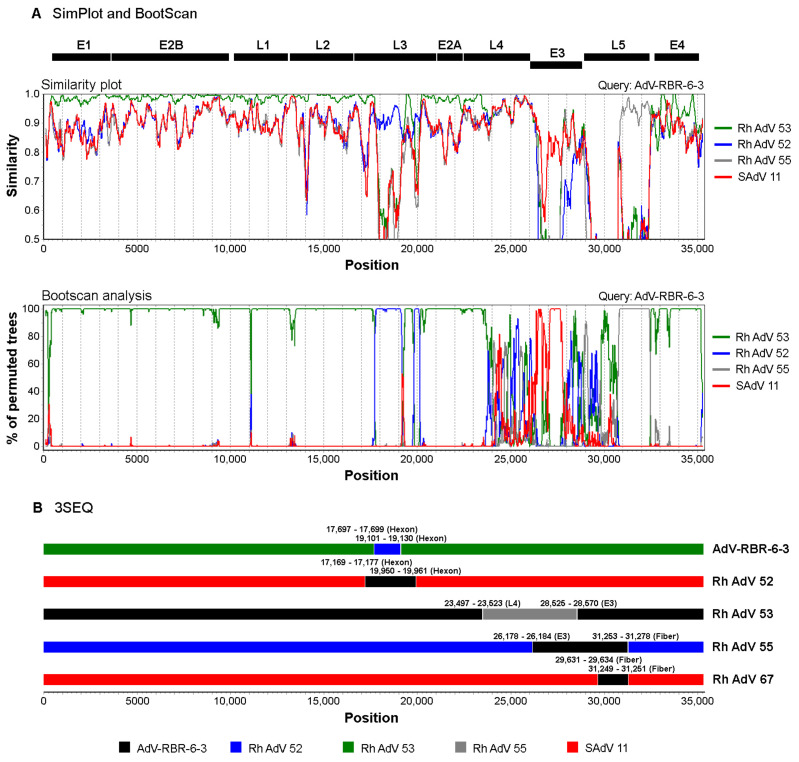
Recombination analyses of AdV-RBR-6-3 complete genome. (**A**) Similarity plot and bootscan analyses to identify potential genetic recombination sites were performed with a sliding window size of 200 nucleotides and a step size of 20 nucleotides using SimPlot. The AdV-RBR-6-3 complete genome was used as a query against reference sequences of *Human adenovirus G*. (**B**) Recombination analysis using 3SEQ suggested recombination events among members of *Human adenovirus G*. Breakpoint intervals and genome regions are indicated for each recombinant. Color labels are indicated for adenovirus sequences. Rh AdV, Rhesus adenovirus; SAdV, Simian adenovirus.

By a BLAST search with the NCBI database, the genome of AdV-RBR-6-3 was found to match with genomes of Rhesus adenoviruses (species *Human mastadenovirus G*) with 92–97% sequence coverage and 89.0–95.9% nucleotide identity. The most closely related genome was that of Rhesus adenovirus 53 with 95.9% nucleotide sequence identity and 96% query coverage. Figure 3A shows phylogenetic analysis using whole genomes. The BLAST result of the AdV-RBR-6-3 complete genome showed that regions of L3 (hexon), E3, and L5 (fiber) were low in sequence identities compared with available adenovirus sequences in the NCBI database (Table S3). The genes involved in DNA synthesis (DNA polymerase), structural genes (penton, hexon, protease, and fiber), and CR1 genes were each analyzed separately. BLASTn showed that DNA polymerase, penton, and protease genes of AdV-RBR-6-3 were closely related to those of Rhesus adenovirus 53 (more than 98% nucleotide sequence identity) (Table 3). However, the hexon, fiber, and CR1 genes were most closely related to those of Rhesus adenovirus 52, Rhesus adenovirus 55, and Simian adenovirus 11, respectively, with nucleotide sequence identities ranging from 71.9–95.3% (Table 3 and Figure 3B–D). The finding suggested that recombination may have occurred in the AdV-RBR-6-3 genome. Similarity plot and bootscan analysis were used to identify a potential recombination in the AdV-RBR-6-3 genome. Recombination sites were identified with breakpoints in L3, E3 and L5 regions (Figure 4A). While a majority of the AdV-RBR-6-3 genome was resembled to the genome of Rhesus adenovirus 53, regions in L3 (hexon), E3 (CR1), and L5 (fiber) were resembled to those of Rhesus adenovirus 52, Simian adenovirus 11, and Rhesus adenovirus 55, respectively. These results were in accordance with the phylogenetic analysis. Recombination analysis using 3SEQ indicated that AdV-RBR-6-3 is a recombinant with potential recombination in the hexon gene (Figure 4B). The virus was also predicted to have experienced multiple recombination events with *Human mastadenovirus G* viruses of simian origin, and potential recombinations in regions of L3 (hexon), L4, E3 (CR1), and L5 (fiber) were identified (Figure 4B and Table S4). Collectively, AdV-RBR-6-3 was isolated from long-tailed macaques in Thailand, suggesting it is a new adenovirus in the species *Human adenovirus G* emerging via intraspecies recombination.

### 3.3. Neutralizing Antibodies against AdV-RBR-6-3 in Long-Tailed Macaques and Humans

**Figure 5:**
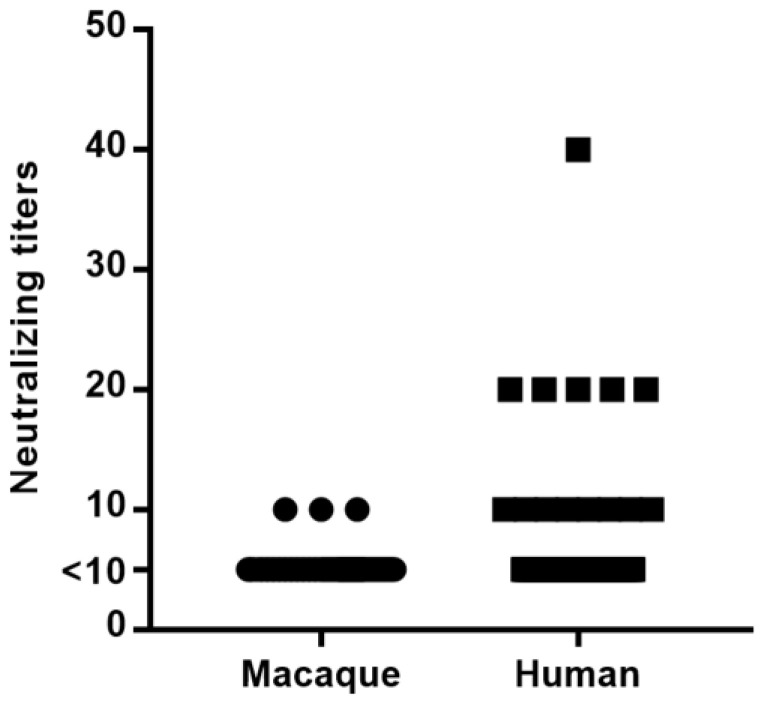
Neutralizing antibody titers against AdV-RBR-6-3 in long-tailed macaque plasmas and human sera in Prachuap Khiri Khan Province. Antibody titer ≥10 was considered as positive.

Neutralizing antibodies against AdV-RBR-6-3 were present in plasmas collected from long-tailed macaques and sera from humans living in the same area of Prachuap Khiri Khan Province (Figure 5). NT antibodies were detected in 2.9% (3/103) of macaque plasmas and 11.2% (14/125) of human sera. In macaques, a low antibody titer of up to 10 was observed, whereas in humans a titer of up to 40 was observed.

## 4. Discussion

Due to its ability to detect genomic sequences of microorganisms in specimens without prior knowledge of genome information, metagenomics has been successfully used for virus detection in disease investigations as mentioned in the Introduction. In those studies, novel viruses were identified on several occasions, showing that the technique has an advantageous power for virus discovery [23,24,25,26]. In this study, VIDISCA metagenomics was used to survey overall virus presence in feces of apparently healthy long-tailed macaques (*M. fascicularis*) in Thailand. It is important for zoonotic surveillance to target healthy populations occasionally, because viral infections may be mostly asymptomatic in their long-term hosts, yet pathogenic in a new host species [15]. Focusing on the eukaryotic virome, the VIDISCA workflow has been developed specifically to identify unknown viruses. Low-speed centrifugation was used to get rid of debris in fecal samples. Nuclease was used to digest host and environmental genome background, preserving intact genomes within virus particles. Non-rRNA hexamers were used as primers to produce an amplified cDNA library. *Mse* I restriction digestion produced appropriate viral genome fragments for further NGS analysis. *Mse* I sites are likely to be present in most viral genomes [20]. For data analysis, the VIDISCA bioinformatics workflow has been designed to target virus sequences. The pipeline identifies “known” and “unknown” virus sequences based on an analysis of VIDISCA fragment sequences against protein and nucleotide databases. Sequencing fragments that find good matches in the protein but not the nucleotide database (aa only) are likely to be derived from unknown or new viruses [21]. We identified VIDISCA sequencing fragments that may have been derived from an unknown astrovirus, enterovirus, or adenovirus in long-tailed macaque feces. The sample size for the metagenomics was relatively small (*n* = 33), and the samples were collected from only one site, which may have limited the representativeness of the findings. The number of sequence reads obtained per sample varied, which may have affected the sensitivity of the virus detection for samples with low reads. Hence, more macaque fecal samples collected in different areas of Thailand were included for PCR confirmation to obtain a wider picture of viral circulation in different areas where macaques and humans live in proximity. Generally, possible pathogens identified by metagenomics should be verified or confirmed by either PCR or isolation [10,18]. Molecular detection suggests the presence of viral genomic sequences in specimens but does not reflect the viability or infectivity of the viruses. Viral isolation confirms the presence of live virus, and the virus can advantageously be used for further studies. Using PCR, we detected the presence of the VIDISCA-suggested sequences from the tentative new astrovirus, enterovirus, and adenovirus in long-tailed macaques in difference areas of Thailand. Potentially novel astroviruses, enteroviruses, and adenoviruses have also been identified and characterized in macaques from other locations [36,37,38]. NHPs have long been recognized as reservoirs of these enteric viruses, and some of them are associated with viruses that cause diseases in humans or have even been proved to have cross-species transmission potential [39,40,41]. We noticed there was variation in the PCR detection rate among the different sampling sites. The detection rate was relatively low in samples from Lopburi and Prachuap Khiri Khan, which were archival samples collected in 2013 and 2017, respectively. The findings may indicate the different shifting patterns of virus distribution among macaque colonies, or it might demonstrate a limitation of using archival samples for molecular detection, which could affect the detection rate. Nevertheless, our findings emphasize a preliminary advantage of using metagenomics in virus discovery and highlight the importance of viral zoonotic surveillance in monkeys. Further molecular, epidemiological, or clinical characterizations of particular viruses identified by metagenomics could provide a deeper understanding of their role in zoonosis. For this purpose, isolation of the virus may be necessarily required. A study designed with comparative groups (healthy vs. disease animals) could also provide a better understanding of the prevalence and the significance of viruses with disease-causing potential.

We isolated one adenovirus (AdV-RBR-6-3) from a long-tailed macaque sample in which VIDISCA suggested the presence of an unknown adenovirus. Confirmation of metagenomics findings by virus isolation is an important step in virus characterization and identification [10]. Hence, it was very fortunate that AdV-RBR-6-3 was successfully isolated. The isolated virus produced positive results in the VIDISCA-based adenovirus PCR. Mass spectrometry analysis also confirmed the isolation of an adenovirus. We noted that the adenovirus species identified by MS (HAdV-F) was not exactly the same as the virus species (HAdV-G) later characterized by whole-genome sequencing, which could have been due to the limited amount of data available in the protein database compared with those in the nucleotide database. Nevertheless, MS has demonstrated its power in aiding virus discovery [29]. HAdV-F and HAdV-G are two adenovirus species that have been associated with gastroenteritis in humans [42]. Phylogenetic analysis of the complete genome (Figure 3A) also gave clues to their molecular evolutionary relationship. Whole-genome analysis classified AdV-RBR-6-3 as a *Human adenovirus G* (HAdV-G) species, together with other adenoviruses previously isolated from rhesus macaques [43,44]. Previously, we reported that different species of adenoviruses circulate in monkeys in Thailand, and HAdV-G is endemic in this area [6]. Adenoviruses are non-enveloped, double-stranded DNA viruses of the genus *Mastadenovirus* in the family *Adenoviridae*. They have icosahedral virions 70–90 nm in diameter and genomes ranging from 26 Kbp to 48 Kbp in size that encode approximately 40 structural and non-structural proteins. There are six genera in the *Adenoviridae* family, namely *Atadenovirus*, *Aviadenovirus*, *Ichtadenovirus*, *Mastadenovirus*, *Siadenovirus*, and *Testadenovirus* [45]. Members of the genus *Mastadenovirus* infect a wide range of mammals, including humans and NHPs, causing diseases that range widely from asymptomatic to respiratory infections, gastroenteritis, conjunctivitis, meningitis, hepatitis, and even systemic or fatal infections. *Mastadenovirus* members currently include >50 species, among which there are seven *Human mastadenovirus* species (HAdV-A to -G) that comprise members isolated from humans and NHPs [45]. The only member of HAdV-G identified from a human is Human adenovirus 52, which was isolated from the stool of a gastroenteritis patient [46]. The other members are of macaque origin (either *M. fascicularis* or *M. mulatta*), including a virus reportedly associated with enteritis [43,44,47]. Evidence of HAdV-G identification and proof of their ability to cause disease in both monkeys and humans suggests that the simian adenoviruses have the potential to cross species barriers and cause disease in humans. Zoonotic surveillance is therefore important to prevent the emergence of new human pathogens. It has been shown in this and the previous studies that adenoviruses isolated from non-human primate hosts were genetically categorized in *Human adenovirus* species based on the current classification. Like HAdV-52 which is the only adenovirus of human host among simian adenoviruses in the HAdV-G species, HAdV-4 is the only member of HAdV-E species identified as a human respiratory pathogen among adenoviruses of chimpanzees [48]. Subsequently, on the taxonomic and phylogenic views, human adenoviruses and simian adenoviruses are suggested to be reclassified using a single designation of “primate adenovirus” (PrAdV) species A to S [49], with the newly characterized AdV-RBR-6-3 classified as a member of the PrAdV-G clade. In this study, AdV-RBR-6-3 was isolated from the feces of an apparently healthy macaque; therefore, we could not find evidence of a clear link between the virus and disease. Further studies involving serological or pathological investigations of the isolated virus are required for a more complete understanding of the role of the virus in human and monkey diseases, as well as its cross-species potential. Subcloning the viral genome and further genome modification could also promote a basic biological understanding of the virus.

Analysis suggests recombination events have occurred in the L3 (hexon), E3 (CR1), and L5 (fiber) regions of the AdV-RBR-6-3 genome, as they differ from those of the most closely related Rhesus adenovirus 53. This is in agreement with the VIDISCA analysis, which identified the CR1 region of AdV-RBR-6-3 as “aa only” or “unknown”. Further recombination analysis showed that AdV-RBR-6-3 is either the parent or recombinant product of multiple recombination events amongst macaque-derived HAdV-G sequences, including Rhesus adenovirus 52, 53, 55, and Simian adenovirus 11 (Table S4). Based on the current information, we could not estimate a timeline or define the order of recombination events within HAdV-G, but the results indicate that intraspecies recombination regularly took place between adenoviruses and could be a source of new circulating viruses. As demonstrated in a study of HAdV-B76, the adenovirus that emerged and caused respiratory disease in humans was the result of multiple intraspecies recombination events among HAdV-B from bonobos, chimpanzees, and humans [8]. Hexon (L3), fiber (L5), and E3 regions are susceptible to recombination, as demonstrated in our work and others’ previous studies [8,42,50,51]. Hexon and fiber are two of the three adenoviral major capsid proteins, and the third is a penton base. Hexon is the most abundant capsid protein, the main building block of the adenovirus capsid, and functions in host cell receptor interaction, viral entry, and tropism determination [52,53]. Fiber is a protruding part containing three structural domains including (1) the tail, which binds to the penton base; (2) the shaft; and (3) the distal knob, which is responsible for binding to host cell surface receptors such as coxsackievirus and adenovirus receptor (CAR), CD46, and sialic acid-containing glycoproteins to initiate viral attachment [52,53]. Different species of adenoviruses contain one to three fibers of different lengths. Members of HAdV-G and many simian adenoviruses have fibers of two different lengths [43,46]. Hexon and fiber are antigenic determinants expressed on the surface of adenoviruses that are subject to neutralizing antibody responses [54]; hence, mutations typically occur at fixed high rates in these genes. As fiber and hexon are involved in receptor binding and virus entry, variations in these proteins could contribute to the emergence of new viruses with altered tropism or host preference. HAdV-D58, a cause of severe chronic diarrhea in AIDS patients, was identified as a novel virus with a unique hexon-coding sequence and a fiber gene resulting from intraspecies recombination [42]. The E3 region of adenoviruses encodes viral proteins involved in the evasion of host immune responses and viral pathogenesis [51]. The E3 transcription units of different human adenoviruses vary in length and the number of open reading frames (ORFs). This region also exhibits high sequence diversity. Based on the complete genome of Human adenovirus 52 (accession no. DQ923122.2), the E3 of HAdV-G contains at least six ORFs, 12.5K, CR1-α, CR1-β, RID-α, RID-β, and 14.7K. In AdV-RBR-6-3, recombination was defined in the CR1 region of E3, the products of which in HAdV-C were identified to play roles in the inhibition of TNF-α-induced apoptosis (CR1-α) and the lysis of infected cells in the viral release step (CR1-β) [51]. The function of this gene in HAdV-G requires further investigation. In HAdV-D, intraspecies homologous recombinations were reported to occur at a high rate in CR1, indicating they play a role in virus evolution [51]. Low nucleotide sequence identities (<85%) of fiber-1 and CR1-α of AdV-RBR-6-3 were also observed, suggesting the occurrence and accumulation of mutation or antigenic drift during the evolution of the virus. Collectively, our work and others’ previous findings emphasize the role of genetic recombination and variation, especially in hexon, fiber, and CR1, in adenovirus evolution. Given that adenoviruses have broad host ranges and tissue tropism and cross-species infections of monkey and human viruses have been described [8,41], continuous collaborative molecular epidemiological, biological, and pathological studies are warranted for zoonotic surveillance to prevent human pathogens emerging.

An investigation of antibody seroprevalence against simian viruses in humans may reveal clues to viral cross-species transmission. It has previously been reported that people who live in the chimpanzee endemic areas in sub-Saharan Africa, where hunting and butchering NHPs for food are widespread and eating bush meat is common, were positive for specific antibodies against chimpanzee adenoviruses at a higher rate compared with people in non-endemic areas [55]. In that study, the seroprevalence rate against three chimpanzee adenoviruses was relatively high in chimpanzees (46% to 92%) and varied between 1.7% and 18.7% in humans living in chimpanzee endemic areas. In the areas included in our study, the hunting and butchering of macaques were not extensively performed. Rather, human–monkey contact possibly occurred during feeding of the wild monkeys or when the monkeys encroached on human habitat [1]. We found that, while AdV-RBR-6-3 was isolated from a macaque, the neutralizing antibody seroprevalence rate of the virus was higher in humans (11.2%) than macaques (2.9%). Serological findings in the macaques seemed to be in agreement with the low detection rate of the virus by the VIDISCA-based PCR (1.1%). It should be noted that the plasma/sera used for neutralizing antibody testing were obtained only from macaques and humans in the Prachuap Khiri Khan site; hence, the results may not represent the overall seroprevalence in monkey and human populations. AdV-RBR-6-3 used in the neutralization assay was isolated from macaque feces collected from Ratchaburi Province. Although we have molecularly confirmed its presence in macaques at the other sites, the PCR confirmation methods utilized only short VIDISCA fragments, which may not exactly represent the whole genome of the virus; the virus was not isolated from samples from the other sites. This could be considered a limitation that restrains the generalization of our findings. Further investigations using a larger sample size collected from different areas may provide a better understanding of the seroprevalence against the macaque viruses. In our previous report, an adenovirus was detected in 41.1% of macaque rectal swabs from Prachuap Khiri Khan using nested PCR specific to adenovirus DNA polymerase [6]. Thirty percent of the detected adenovirus sequences were of HAdV-G, which is closely related to Rhesus adenoviruses. It is possible that the neutralizing seropositive properties of human sera in Prachuap Khiri Khan were a result of cross-neutralization with the other macaque-borne HAdV-G. In a study in which pre-existing antibodies against several adenovirus genotypes were tested, high neutralizing reactivity and seropositivity against various adenoviruses, i.e., HAdV-B, HAdV-C, HAdV-E, HAdV-G, and HAdV-F, were observed in human sera [56]. Pre-existing adenoviral antibodies could be a source of cross-neutralization. As hexon is a major target for neutralizing antibodies, more studies may be required, including those focusing on the role of the genetic recombination in hexon that results in antigenic variation and, hence, antibody responses. Although we could not come to a firm conclusion about cross-neutralization using the present data, mainly sero-surveillance evidence from Prachuap Khiri Khan alone, cross-species infection by adenoviruses in monkeys and humans was suggested to occur. Although more investigations are required to confirm and elucidate the transmission direction, the findings emphasize the possibility of the cross-species transmission of adenoviruses between humans and monkeys and the importance of zoonotic surveillance, especially in areas where humans and animals share habitats.

Regarding the use of adenoviruses as vectors for gene therapy and vaccine delivery, pre-existing antibodies that can neutralize or cross-neutralize the vectors are a concern, as these factors could compromise the gene transfer efficacy. It is notable that cross-neutralizing antibodies may occur as a result of repeated infections [54], and so precaution should also be taken when selecting adenovirus vectors for the development of adenovirus vector-based vaccines, especially those used in populations living in proximity to monkeys.

## 5. Conclusions

VIDISCA metagenomics successfully detected an unknown adenovirus in an asymptomatic long-tailed macaque in Thailand. The isolation of adenovirus AdV-RBR-6-3 led to the classification and molecular characterization of a novel HAdV-G from monkeys which emerged as a result of genetic recombination among macaque adenoviruses. A serological study of neutralizing antibodies in humans and monkeys indicated infection by the macaque-borne adenovirus in humans. Although we could not decisively conclude the transmission direction, whether zoonosis or zooanthroponosis, the finding indicates that pathogen surveillance is important and should be continued, especially in areas where humans and animals interact, to predict and prevent emerging of new pathogens based upon the One Health concept of human–animal–environmental health.

## Figures and Tables

**Table 1:** Primer sequences.

Virus	Primer	PCR Round	Sequence (5′–3′)	Annealing Temp. (°C)	Expected Product Size	Gene
Adenovirus	Adeno F	First	GCTCGCCATCATTCCAGTTC	50	212	CR1
	Adeno R	First	CTGAGTAGAGTTTGGGAGTTG			
	Adeno FN	Nested	CCAGTTCTTTACTTAGCCTTGC	50	171	CR1
	Adeno RN	Nested	ACGTTTAGTTCCTACTTGAGC			
Enterovirus	Entero F	First	ACACATCGTGAAACACACTG	55	312	Protease
	Entero R	First	GTTGTACCAATCGTGATGTTC			
	Entero FN	Nested	ATCCATTGTATCTCCCCTTG	55	185	Protease
	Entero RN	Nested	GTGGAAAGCCCACCCATAGA			
Astrovirus	Astro F	First	TGCCAATTATGCGGCTTCTC	50	312	Capsid
	Astro R	First	GTGATGGTAAATGTTCTAGAC			
	Astro FN	Nested	TACTAAGACACTGGCTATTGC	50	98	Capsid
	Astro RN	Nested	TTCCGCCATACTGAGATTTC			

**Table 2:** Detection of putative unknown virus sequences by PCR or RT-PCR with VIDISCA primer in long-tailed macaques.

Site	Sample Type	Collection Year	No. of Samples	No. of Positives (%)		
				AstV	EV	AdV
Ratchaburi	Feces	2018–2019	39 *	4 (10.3%)	5 (12.8%)	5 (12.8%)
Kanchanaburi	Feces	2018–2019	12	0	0	2 (16.7%)
Lopburi	Feces	2013	46	0	9 (19.6%)	1 (2.2%)
Prachuap Khiri Khan	Rectal swab	2017	90	2 (2.2%)	0	1 (1.1%)
Total			187	6 (3.2%)	14 (7.5%)	9 (4.8%)

* Among 39 fecal samples from Ratchaburi, 33 were subjected for VIDISCA NGS analysis. AstV, astrovirus; EV, enterovirus; AdV, adenovirus.

**Table 3:** Percent nucleotide sequence identity of AdV-RBR-6-3 genes with adenovirus sequences in NCBI database.

Gene	Transcription Class	Sequence Coverage	% Identity	Most Identical AdV	Accession No.
DNA polymerase	E2B	100%	98.7%	Rhesus AdV 53	KM591903.1
III (penton base)	L2	100%	99.3%	Rhesus AdV 53	KM591903.1
II (hexon)	L3	100%	91.6%	Rhesus AdV 52	KM591902.1
Protease	L3	100%	98.9%	Rhesus AdV 53	KM591903.1
Fiber-1	L5	98%	71.9%	Rhesus AdV 55	MF198449.1
Fiber-2	L5	100%	95.3%	Rhesus AdV 55	MF198449.1
CR1-α	E3	100%	83.6%	Simian AdV 11	KP329562.1
CR1-β	E3	99%	85.0%	Simian AdV 11	KP329562.1

AdV, adenovirus.

## Data Availability

Data are contained within the article and Supplementary Material. The whole-genome sequence of AdV-RBR-6-3 was submitted to GenBank (https://www.ncbi.nlm.nih.gov/genbank/) and received accession no. OQ579036, accessed on 1 June 2023.

## Supplementary Materials

The following supporting information can be downloaded at: https://www.mdpi.com/1999-4915/15/6/1371/s1, Table S1: BLAST results of VIDISCA-based PCR products; Table S2: Sequences of primers used for PCR to fill gaps in AdV-RBR-6-3 whole genome; Table S3: Alignment of AdV-RBR-6-3 genome (Query 64407) with other adenoviruses; Table S4: Result of recombination analysis analyzed by 3SEQ v1.7 software.
