# Correction: Kosoltanapiwat et al. A Novel Simian Adenovirus Associating with Human Adenovirus Species G Isolated from Long-Tailed Macaque Feces. *Viruses* 2023, *15*, 1371

**DOI:** 10.3390/v15091871

**Published:** 2023-09-04

**Authors:** Nathamon Kosoltanapiwat, Lia van der Hoek, Cormac M. Kinsella, Jarinee Tongshoob, Luxsana Prasittichai, Michelle Klein, Maarten F. Jebbink, Martin Deijs, Onrapak Reamtong, Kobporn Boonnak, Wathusiri Khongsiri, Juthamas Phadungsombat, Daraka Tongthainan, Phitsanu Tulayakul, Marnoch Yindee

**Affiliations:** 1 Department of Microbiology and Immunology, Faculty of Tropical Medicine, Mahidol University, Bangkok 10400, Thailand; 2 Amsterdam UMC, Laboratory of Experimental Virology, Department of Medical Microbiology and Infection Prevention, University of Amsterdam, 1105 AZ Amsterdam, The Netherlands; 3 Wildlife Conservation Division, Protected Areas Regional Office 3 (Ban Pong), Department of National Parks, Wildlife and Plant Conservation, Ministry of Natural Resources and Environment, Ratchaburi 70110, Thailand; 4 Department of Molecular Tropical Medicine and Genetics, Faculty of Tropical Medicine, Mahidol University, Bangkok 10400, Thailand; 5 Department of Immunology, Faculty of Medicine Siriraj Hospital, Mahidol University, Bangkok 10700, Thailand; 6 Department of Viral Infections, Research Institute for Microbial Diseases, Osaka University, Osaka 565-0871, Japan; 7 Faculty of Veterinary Medicine, Rajamangala University of Technology Tawan-ok, Chonburi 20110, Thailand; 8 Department of Veterinary Public Health, Faculty of Veterinary Medicine, Kasetsart University, Nakhon Pathom 73140, Thailand; 9 Akkhraratchakumari Veterinary College, Walailak University, Nakhonsithammarat 80161, Thailand

After publication of the article, the authors received comments from a member of the *Viruses* editorial board who is an expert in the field of adenovirus concerning figures and references that should be included in the paper. In response to those suggestions, the authors wish to make the following corrections to this paper [1]:

## Replacement of Figure

First, there is the replacement of Figure 3 to include more reference sequences in the phylogenetic trees. The corrected Figure 3 appears below.

With regard to the replacement of Figure 3, updates are made in three places: 

(1) the Figure 3 legend; 

The sentence “*Simian adenovirus A* and *B*, *Human adenovirus F*, and *Human adenovirus G*. Black triangle indicates AdV-RBR-6-3.” is replaced by “*Human adenovirus A* to *G* (HAdV-A to -G), *Simian adenovirus A* (SAdV-A) and *B* (SAdV-B). Black triangle indicates AdV-RBR-6-3. Diamond indicates Human adenovirus 52, the only member of species HAdV-G isolated from human. Tree shrew adenovirus sequences were used as an outgroup for the whole genome and hexon trees. SAdV-A sequences were used as an outgroup for the fiber-1 and CR1 trees. According to variabilities in fiber and CR1 regions amongst mastadenoviruses, sequences with ≥30% identities were included in the trees.”.

(2) in Materials and Methods, the first paragraph of Section 2.7;

To provide information about the phylogenetic models used to create the phylogenetic trees in the updated Figure 3, the sentences “The general time reversible model was used for the complete genome. The Tamura–Nei model was used for the hexon gene. The Hasegawa–Kishino–Yano model was used for fiber-1 and CR1 genes. Bootstrap resampling analysis of 1000 replicates was used.” are changed to “The general time reversible model was used for the complete genome, hexon and CR1. The Hasegawa–Kishino–Yano model was used for fiber-1. Bootstrap resampling analysis of 1000 replicates was used.”.

(3) in the Discussion, the second paragraph;

The sentence “Phylogenetic analysis of the hexon gene (Figure 3B) also gave clues to their molecular evolutionary relationship.” is replaced by “Phylogenetic analysis of the complete genome (Figure 3A) also gave clues to their molecular evolutionary relationship”; The sentences “It has been shown in this and the previous studies that adenoviruses isolated from non-human primate hosts were genetically categorized in Human adenovirus species based on the current classification. Like HAdV-52 which is the only adenovirus of human host among simian adenoviruses in the HAdV-G species, HAdV-4 is the only member of HAdV-E species identified as a human respiratory pathogen among adenoviruses of chimpanzees [48]. Subsequently, on the taxonomic and phylogenic views, human adenoviruses and simian adenoviruses are suggested to be reclassified using a single designation of “primate adenovirus” (PrAdV) species A to S [49], with the newly characterized AdV-RBR-6-3 classified as a member of the PrAdV-G clade.” are added.

## Insertion of Figure

Next, there is the insertion of a new Figure 4 to illustrate the recombination that occurred in the adenovirus genomes. The corrected Figure 4 and its legend appear below.

With regard to the insertion of the new Figure 4 and its legend, updates are made in three places: 

(1) in Materials and Methods, the second paragraph of Section 2.7;

To provide more information about the methods used for recombination analyses, the sentences “Recombination analysis was performed with the complete AdV-RBR-6-3 genome and simian adenovirus genomes retrieved from GenBank using 3SEQ v1.7 [33]. Sequences were aligned, and all sequence triplets were tested using a non-parametric statistic for mosaicism.” are replaced by “Recombination analyses were performed with the complete AdV-RBR-6-3 genome and simian adenovirus genomes retrieved from GenBank using two software, SimPlot v3.5.1 [33] and 3SEQ v1.7 [34]. The sequences were aligned using ClustalW in BioEdit. Similarity plot and bootscan analyses were performed by SimPlot using default parameter settings. The Kimura 2-parameter distance model, in a sliding window of 200 nucleotides, and step size of 20 nucleotides was applied for the similarity plot, and the Kimura 2-parameter distance model and neighbor-joining tree model, in a sliding window of 200 nucleotides, and step size of 20 nucleotides were used for bootscan analysis. In 3SEQ, sequences were aligned, and all sequence triplets were tested using a non-parametric statistic for mosaicism.”.

(2) in the Results, the third paragraph of Section 3.2;

To provide a text description to the updated Figure 4, we add the sentences “Similarity plot and bootscan analysis were used to identify a potential recombination in the AdV-RBR-6-3 genome. Recombination sites were identified with breakpoints in L3, E3 and L5 regions (Figure 4A). While a majority of the AdV-RBR-6-3 genome was resembled to the genome of Rhesus adenovirus 53, regions in L3 (hexon), E3 (CR1), and L5 (fiber) were resembled to those of Rhesus adenovirus 52, Simian adenovirus 11, and Rhesus adenovirus 55, respectively. These results were in accordance with the phylogenetic analysis.”; Figure 4B is cited in the sentences “Recombination analysis using 3SEQ indicated that AdV-RBR-6-3 is a recombinant with potential recombination in the hexon gene (Figure 4B). The virus was also predicted to have experienced multiple recombination events with Human mastadenovirus G viruses of simian origin, and potential recombinations in regions of L3 (hexon), L4, E3 (CR1), and L5 (fiber) were identified (Figure 4B and Table S4).”.

(3) the former Figure 4 is renumbered to Figure 5.

## Insertion of Citation

In the original publication, “Lole, K.S.; Bollinger, R.C.; Paranjape, R.S.; Gadkari, D.; Kulkarni, S.S.; Novak, N.G.; Ingersoll, R.; Sheppard, H.W.; Ray, S.C. Full-length human immunodeficiency virus type 1 genomes from subtype C-infected seroconverters in India, with evidence of intersubtype recombination. *J. Virol.* **1999**, *73*, 152–160.”, “Purkayastha, A.; Ditty, S.E.; Su, J.; McGraw, J.; Hadfield, T.L.; Tibbetts, C.; Seto, D. Genomic and bioinformatics analysis of HAdV-4, a human adenovirus causing acute respiratory disease: implications for gene therapy and vaccine vector development. *J. Virol.* **2005**, *79*, 2559–2572.” and “Kang, J.; Ismail, A.M.; Dehghan, S.; Rajaiya, J.; Allard, M.W.; Lim, H.C.; Dyer, D.W.; Chodosh, J.; Seto, D. Genomics-based re-examination of the taxonomy and phylogeny of *human* and *simian Mastadenoviruses*: an evolving whole genomes approach, revealing putative zoonosis, anthroponosis, and amphizoonosis. *Cladistics* **2020**, *36*, 358–373.” were not cited. The citations have now been inserted in the second paragraph of Section 2.7 and second paragraph of the Discussion and should read:

“Recombination analyses were performed with the complete AdV-RBR-6-3 genome and simian adenovirus genomes retrieved from GenBank using two software, SimPlot v3.5.1 [33] and 3SEQ v1.7”.

“It has been shown in this and the previous studies that adenoviruses isolated from non-human primate hosts were genetically categorized in *Human adenovirus* species based on the current classification. Like HAdV-52 which is the only adenovirus of human host among simian adenoviruses in the HAdV-G species, HAdV-4 is the only member of HAdV-E species identified as a human respiratory pathogen among adenoviruses of chimpanzees [48]. Subsequently, on the taxonomic and phylogenic views, human adenoviruses and simian adenoviruses are suggested to be reclassified using a single designation of “primate adenovirus” (PrAdV) species A to S [49], with the newly characterized AdV-RBR-6-3 classified as a member of the PrAdV-G clade.”.

With this correction, the order of some references has been adjusted accordingly. The authors state that the scientific conclusions are unaffected. This correction was approved by the Academic Editor. The original publication has also been updated.

## Figures and Tables

**Figure 3:**
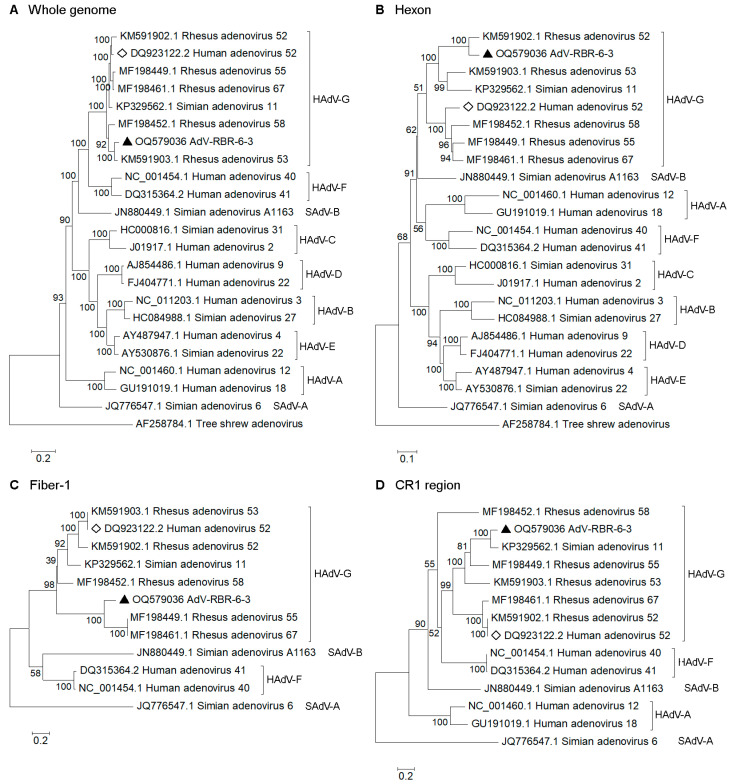
Phylogenetic analysis of (**A**) whole genome, (**B**) hexon, (**C**) fiber-1, and (**D**) CR1 genes of AdV-RBR-6-3 compared with reference sequences of *Human adenovirus A* to *G* (HAdV-A to -G), *Simian adenovirus A* (SAdV-A) and *B* (SAdV-B). Black triangle indicates AdV-RBR-6-3. Diamond indicates Human adenovirus 52, the only member of species HAdV-G isolated from human. Tree shrew adenovirus sequences were used as an outgroup for the whole genome and hexon trees. SAdV-A sequences were used as an outgroup for the fiber-1 and CR1 trees. According to variabilities in fiber and CR1 regions amongst mastadenoviruses, sequences with ≥30% identities were included in the trees. The trees were constructed using the maximum likelihood method with a bootstrap of 1000. Bootstrap values are shown at the node. The bar represents nucleotide substitutions per site.

**Figure 4:**
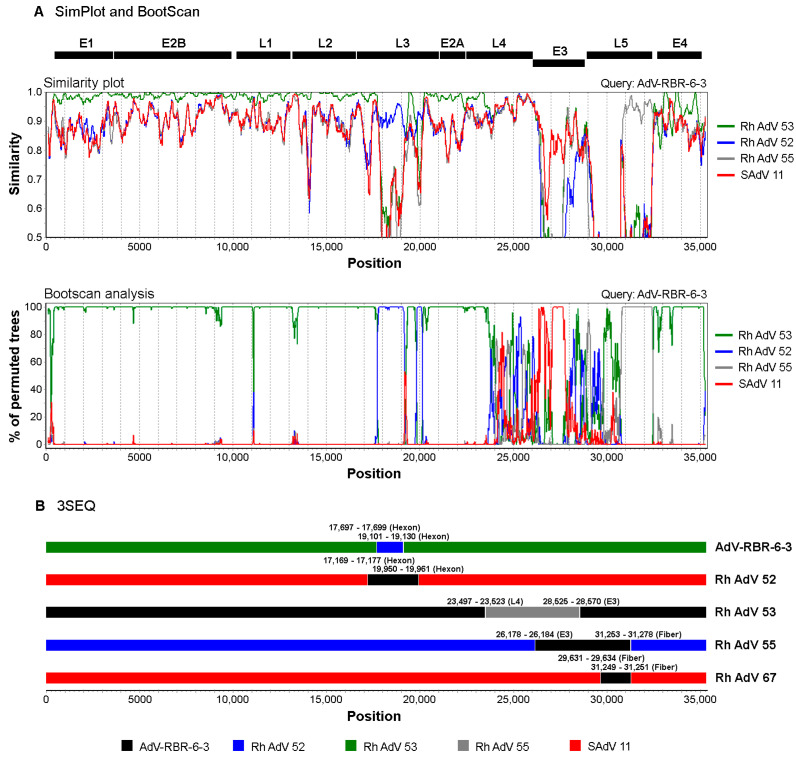
Recombination analyses of AdV-RBR-6-3 complete genome. (**A**) Similarity plot and bootscan analyses to identify potential genetic recombination sites were performed with a sliding window size of 200 nucleotides and a step size of 20 nucleotides using SimPlot. The AdV-RBR-6-3 complete genome was used as a query against reference sequences of *Human adenovirus G*. (**B**) Recombination analysis using 3SEQ suggested recombination events among members of *Human adenovirus G*. Breakpoint intervals and genome regions are indicated for each recombinant. Color labels are indicated for adenovirus sequences. Rh AdV, Rhesus adenovirus; SAdV, Simian adenovirus.
