# Supplementary material for: A Novel Simian Adenovirus Associating with Human Adeno-virus Species G Isolated from Long-Tailed Macaque Feces
Source: Viruses. 2023 Jun 14;15(6):1371. doi: 10.3390/v15061371 (PMC10303043; doi:10.3390/v15061371)
Supplement: Supplementary file 1 [file viruses-15-01371-s001.zip › Table S1.pdf]

Table S1. BLAST results of VIDISCA-based PCR products.

Virus	Collection site	Sample ID	Best match by BLASTn	Description	% query coverage	% nucleotide identity
Astrovirus (85 bp)	Ratchaburi	6-5	BR001717.1	Macaque MLB-like astrovirus	100%	83.53%
		7-1	BR001717.1	Macaque MLB-like astrovirus	100%	83.53%
		7-12	BR001717.1	Macaque MLB-like astrovirus	100%	83.53%
		7-16	BR001717.1	Macaque MLB-like astrovirus	100%	83.53%
	Prachuap Khiri Khan	H49	BR001717.1	Macaque MLB-like astrovirus	100%	83.53%
		H51	BR001717.1	Macaque MLB-like astrovirus	100%	83.53%
Enterovirus (163 bp)	Ratchaburi	6-5	KT961650.1	Simian enterovirus A92	99%	87.65%
		7-3	KT961655.1	Enterovirus A124	98%	88.20%
		7-10	KT961650.1	Simian enterovirus A92	99%	86.42%
		7-15	KT961655.1	Enterovirus A124	98%	88.20%
		7-16	KT961655.1	Enterovirus A124	98%	88.20%
	Lopburi	M1-5	MT649088.1	Simian enterovirus 19	100%	82.21%
		M1-9	MT649088.1	Simian enterovirus 19	100%	82.82%
		M1-13	MT649088.1	Simian enterovirus 19	100%	82.82%
		M3-8	OK397297.1	Enterovirus A	100%	83.44%
		M3-9	OK397297.1	Enterovirus A	100%	84.05%
		M3-11	OK397297.1	Enterovirus A	100%	84.05%
		M4-4	MT649088.1	Simian enterovirus 19	100%	82.21%
		M4-5	MT649088.1	Simian enterovirus 19	100%	84.05%
		M5-1	MT649088.1	Simian enterovirus 19	100%	82.21%
Adenovirus (147 bp)	Ratchaburi	6-3	OQ579036.1	HAdV-G isolate AdV-RBR-6-3	100%	100%
		6-9	OQ579036.1	HAdV-G isolate AdV-RBR-6-3	100%	100%
		7-5	OQ579036.1	HAdV-G isolate AdV-RBR-6-3	100%	100%
		7-9	OQ579036.1	HAdV-G isolate AdV-RBR-6-3	100%	100%
		7-14	OQ579036.1	HAdV-G isolate AdV-RBR-6-3	100%	100%
	Kanchanaburi	1-9	OQ579036.1	HAdV-G isolate AdV-RBR-6-3	100%	100%
		1-10	OQ579036.1	HAdV-G isolate AdV-RBR-6-3	100%	100%
	Lopburi	M5-11	OQ579036.1	HAdV-G isolate AdV-RBR-6-3	100%	100%
	Prachuap Khiri Khan	H27	OQ579036.1	HAdV-G isolate AdV-RBR-6-3	100%	100%
