# Supplementary material for: A Novel Simian Adenovirus Associating with Human Adeno-virus Species G Isolated from Long-Tailed Macaque Feces
Source: Viruses. 2023 Jun 14;15(6):1371. doi: 10.3390/v15061371 (PMC10303043; doi:10.3390/v15061371)
Supplement: Supplementary file 1 [file viruses-15-01371-s001.zip › Table S2.pdf]

Table S2. Sequences of primers used for PCR to fill gaps in AdV-RBR-6-3 whole genome.

Primer	Sequence 5'-3'	Position on AdV-RBR-6-3[a]
P1-F	CCTTATTCTGGAAACGTGCCAA	17-38
P1-R	TAAATGGGACCTTTGAGCCTTC	323-344
P2-F	CGATGTAACCACGGACCGCTC	17625-17645
P2-R	GTTGTGGGCCATGGGGAAGAAG	19197-19218
P3-F	ACAAGGACAGAATGTACTCCTTCT	19703-19726
P3-R	AGCGGAGTTAGCGTACAGCA	20018-20037
P4-F	CTACTTTGAGCTGCCGGACG	25933-25952
P4-R	CTGGTAATGGCTGTRCAGACCA	27635-27656
P5-F	TGGTCTGTACAGCCATTACCAG	27635-27656
P5-R	CGCCCACCATCATATCCCATATG	28560-28582
P6-F	CATATGGGATATGATGGTGGGCG	28560-28582
P6-R	CAGAAGTTTACCGCCATGAAGG	31323-31344
P7-F	CAGCCATTGCCCCGTCTTAC	33782-33801
P7-R	TGATAATGAGTGGGGAGGAGC	34084-34104

[a]AdV-RBR-6-3 accession no. OQ579036
