# Supplementary material for: A Novel Simian Adenovirus Associating with Human Adeno-virus Species G Isolated from Long-Tailed Macaque Feces
Source: Viruses. 2023 Jun 14;15(6):1371. doi: 10.3390/v15061371 (PMC10303043; doi:10.3390/v15061371)
Supplement: Supplementary file 1 [file Table S3.pdf › Table S3.pdf]

Table S3. Alignment of AdV-RBR-6-3 genome (Query 64407) with other adenoviruses.

NCBI Multiple Sequence Alignment Viewer, Version 1.23.1

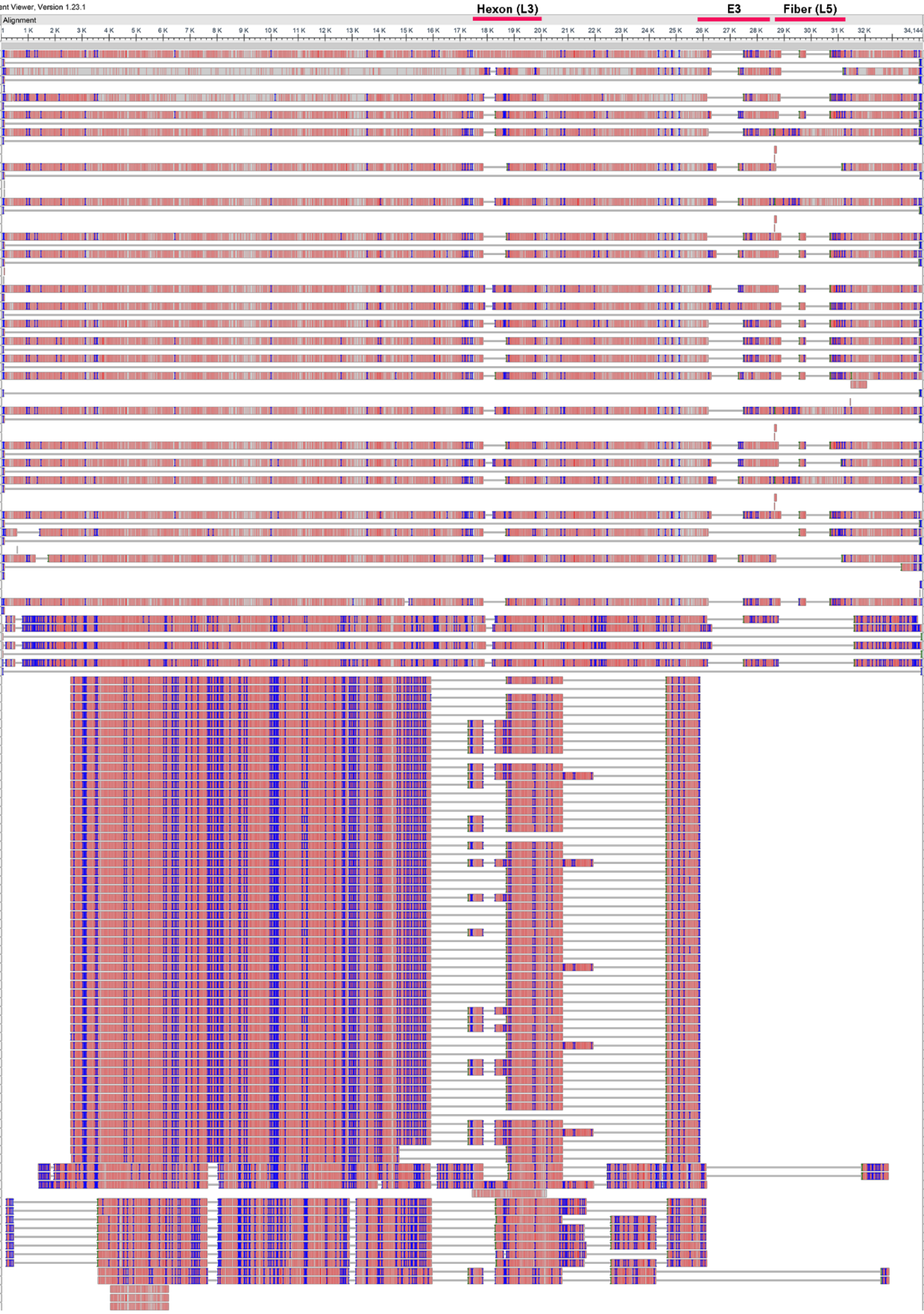

Sequence ID		Start	End	Organism
Query_64407	(+)	1	34,144	
KM591902.1	(+)	1	34,391	Rhesus adenovirus 52
KM591902.1	(-)	34,391	1	Rhesus adenovirus 52
KM591903.1	(+)	1	34,475	Rhesus adenovirus 53
KM591903.1	(-)	34,475	1	Rhesus adenovirus 53
KM591903.1	(+)	95	108	Rhesus adenovirus 53
MF198452.1	(+)	1	34,364	Rhesus adenovirus 58
MF198452.1	(-)	34,364	1	Rhesus adenovirus 58
MF198458.1	(+)	1	35,213	Rhesus adenovirus 64
MF198458.1	(-)	35,213	1	Rhesus adenovirus 64
MF198461.1	(+)	1	35,125	Rhesus adenovirus 67
MF198461.1	(-)	35,125	1	Rhesus adenovirus 67
MF198461.1	(+)	28,753	28,811	Rhesus adenovirus 67
MF198461.1	(+)	29,614	29,622	Rhesus adenovirus 67
MF198455.1	(+)	1	35,192	Rhesus adenovirus 61
MF198455.1	(-)	35,192	1	Rhesus adenovirus 61
MF198455.1	(+)	131	136	Rhesus adenovirus 61
MF198455.1	(+)	100	104	Rhesus adenovirus 61
MF198453.1	(+)	1	35,126	Rhesus adenovirus 59
MF198453.1	(-)	35,126	1	Rhesus adenovirus 59
MF198453.1	(+)	28,753	28,811	Rhesus adenovirus 59
MF198453.1	(+)	29,615	29,623	Rhesus adenovirus 59
MF198448.1	(+)	1	34,433	Rhesus adenovirus 54
MF198448.1	(-)	34,433	1	Rhesus adenovirus 54
MF198459.1	(+)	1	35,252	Rhesus adenovirus 65
MF198459.1	(-)	35,252	1	Rhesus adenovirus 65
MF198459.1	(+)	94	112	Rhesus adenovirus 65
MF198459.1	(+)	126	128	Rhesus adenovirus 65
MF198460.1	(+)	1	35,158	Rhesus adenovirus 66
MF198460.1	(-)	35,158	35,055	Rhesus adenovirus 66
KP329562.1	(+)	1	34,510	Simian adenovirus 11
KP329562.1	(-)	34,510	1	Simian adenovirus 11
MF198456.1	(+)	1	34,466	Rhesus adenovirus 62
MF198456.1	(-)	34,466	1	Rhesus adenovirus 62
NC_006879.1	(+)	1	34,450	Simian adenovirus 1
NC_006879.1	(-)	34,450	1	Simian adenovirus 1
AY771780.1	(+)	1	34,450	Simian adenovirus 1
AY771780.1	(-)	34,450	1	Simian adenovirus 1
KM591901.1	(+)	1	35,079	Rhesus adenovirus 51
KM591901.1	(+)	31,847	32,424	Rhesus adenovirus 51
KM591901.1	(-)	35,079	1	Rhesus adenovirus 51
KM591901.1	(+)	32,429	32,447	Rhesus adenovirus 51
MF198451.1	(+)	1	35,094	Rhesus adenovirus 57
MF198451.1	(-)	35,094	1	Rhesus adenovirus 57
MF198451.1	(+)	28,721	28,779	Rhesus adenovirus 57
MF198451.1	(+)	29,583	29,591	Rhesus adenovirus 57
MF198454.1	(+)	1	35,182	Rhesus adenovirus 60
MF198454.1	(-)	35,182	1	Rhesus adenovirus 60
MF198457.1	(+)	1	35,052	Rhesus adenovirus 63
MF198457.1	(-)	35,052	1	Rhesus adenovirus 63
MF198449.1	(+)	1	35,178	Rhesus adenovirus 55
MF198449.1	(-)	35,178	1	Rhesus adenovirus 55
MF198449.1	(+)	28,813	28,871	Rhesus adenovirus 55
MF198449.1	(+)	29,675	29,683	Rhesus adenovirus 55
MF198450.1	(+)	1	34,449	Rhesus adenovirus 56
MF198450.1	(-)	34,449	1	Rhesus adenovirus 56
DQ792570.1	(+)	1	31,045	Simian adenovirus 7
DQ792570.1	(-)	31,045	1	Simian adenovirus 7
DQ792570.1	(+)	610	612	Simian adenovirus 7
MG969551.1	(+)	1	35,481	Simian adenovirus 2
MG969551.1	(-)	35,481	1	Simian adenovirus 2
MG969551.1	(+)	1,299	1,411	Simian adenovirus 2
MG969551.1	(-)	1,395	1,299	Simian adenovirus 2
MG969551.1	(-)	104	97	Simian adenovirus 2
DQ923122.2	(+)	1	34,250	Human adenovirus 52
DQ923122.2	(-)	34,250	34,138	Human adenovirus 52
NC_021168.1	(+)	170	34,264	Baboon adenovirus 3
KP329565.1	(+)	1	34,064	Simian adenovirus 19
KP329565.1	(-)	34,064	1	Simian adenovirus 19
NC_028107.1	(+)	1	34,064	Simian adenovirus 19
NC_028107.1	(-)	34,064	1	Simian adenovirus 19
KC693023.1	(+)	1	34,402	Simian mastadenovirus C
KC693023.1	(-)	34,402	1	Simian mastadenovirus C
ON442323.1	(+)	2,652	26,124	Human adenovirus 41
MK962807.1	(+)	2,644	26,138	Human mastadenovirus F
DQ315364.2	(+)	2,655	26,148	Human adenovirus 41
ON442325.1	(+)	2,656	26,140	Human adenovirus 41
OP047717.1	(+)	2,671	26,130	Human adenovirus 41
ON442321.1	(+)	2,653	26,153	Human adenovirus 41
ON442329.1	(+)	2,640	26,141	Human adenovirus 41
ON442317.1	(+)	2,649	26,148	Human adenovirus 41
ON442312.1	(+)	2,640	26,141	Human adenovirus 41
MG925783.1	(+)	2,642	26,139	Human adenovirus 41
ON442313.1	(+)	2,646	26,150	Human adenovirus 41
MW567962.1	(+)	2,582	26,083	Human adenovirus 41
KY316160.1	(+)	2,654	26,143	Human adenovirus 41
OP174925.1	(+)	2,640	26,122	Human adenovirus 41
OP174923.1	(+)	2,589	26,071	Human adenovirus 41
OP174918.1	(+)	2,640	26,121	Human adenovirus 41
MH465394.1	(+)	2,651	26,138	Human adenovirus 41
ON442330.1	(+)	2,682	26,174	Human adenovirus 41
MW567963.1	(+)	2,587	26,090	Human adenovirus 41
HM565136.3	(+)	2,657	26,143	Human adenovirus 41
OP174924.1	(+)	2,637	26,130	Human adenovirus 41
OP174922.1	(+)	2,644	26,147	Human adenovirus 41
OP047706.1	(+)	2,649	26,142	Human adenovirus 41
ON532823.1	(+)	2,656	26,146	Human adenovirus 41
ON442327.1	(+)	2,643	26,138	Human adenovirus 41
MZ546190.1	(+)	2,594	26,100	unidentified adenovirus
MW567966.1	(+)	2,576	26,071	Human adenovirus 41
MW567965.1	(+)	2,576	26,071	Human adenovirus 41
MW567964.1	(+)	2,576	26,071	Human adenovirus 41
KY316162.1	(+)	2,657	26,139	Human adenovirus 41
OP047716.1	(+)	2,642	26,136	Human adenovirus 41
OP047715.1	(+)	2,647	26,141	Human adenovirus 41
OP047714.1	(+)	2,643	26,137	Human adenovirus 41
OP174917.1	(+)	2,641	26,132	Human adenovirus 41
OP047705.1	(+)	2,648	26,141	Human adenovirus 41
ON532827.1	(+)	2,648	26,147	Human adenovirus 41
ON532821.1	(+)	2,650	26,145	Human adenovirus 41
ON442324.1	(+)	2,642	26,135	Human adenovirus 41
KX868523.2	(+)	2,639	26,144	Human adenovirus 41
ON532820.1	(+)	2,655	26,142	Human adenovirus 41
ON532818.1	(+)	2,655	26,160	Human adenovirus 41
OP047707.1	(+)	2,663	26,139	Human adenovirus 41
OP047700.1	(+)	2,646	26,137	Human adenovirus 41
ON442328.1	(+)	2,648	26,138	Human adenovirus 41
ON442320.1	(+)	2,652	26,158	Human adenovirus 41
ON442319.1	(+)	2,652	26,158	Human adenovirus 41
ON561778.1	(+)	2,650	26,143	Human adenovirus 41
OP174920.1	(+)	2,642	26,145	Human adenovirus 41
OP174919.1	(+)	2,642	26,145	Human adenovirus 41
OP174916.1	(+)	2,642	26,145	Human adenovirus 41
MK962806.1	(+)	2,642	26,136	Human mastadenovirus F
ON532819.1	(+)	2,652	26,155	Human adenovirus 41
OP174921.1	(+)	2,632	26,138	Human adenovirus 41
ON442314.1	(+)	2,649	26,145	Human adenovirus 41
OP047712.1	(+)	2,647	26,137	Human adenovirus 41
OP047719.1	(+)	2,672	26,142	Human adenovirus 41
NC_022266.1	(+)	1,913	30,694	Simian adenovirus 18
FJ025931.1	(+)	1,913	30,694	Simian adenovirus 18
KP329566.1	(+)	1,632	26,766	Simian adenovirus 17
KF053126.1	(+)	1	2,739	Simian adenovirus KRF...
KT013209.1	(+)	276	26,968	Cynomolgus adenovirus 1
NC_034382.1	(+)	276	26,968	Cynomolgus adenovirus 1
JN880456.1	(+)	284	26,960	Simian adenovirus A1335
JN880455.1	(+)	283	26,945	Simian adenovirus A1327
JN880454.1	(+)	283	26,944	Simian adenovirus A1312
JN880453.1	(+)	262	26,953	Simian adenovirus A1296
JN880448.1	(+)	284	26,979	Simian adenovirus A1139
KC693021.1	(+)	283	26,959	Simian adenovirus B
KP329564.1	(+)	3,919	33,859	Simian adenovirus 16
NC_028105.1	(+)	3,919	33,859	Simian adenovirus 16
KP853123.1	(+)	2	2,154	Simian adenovirus 12
KP853120.1	(+)	2	2,154	Simian adenovirus 2
KP853109.1	(+)	2	2,154	Simian adenovirus 15
